# Prognosis and clinical characteristics of patients with 3β-hydroxy-Δ5-C27-steroid dehydrogenase deficiency diagnosed in childhood: A systematic review of the literature

**DOI:** 10.1097/MD.0000000000028834

**Published:** 2022-02-18

**Authors:** Yuan Zhang, Chun-Feng Yang, Wen-Zhen Wang, Yong-Kang Cheng, Chu-Qiao Sheng, Yu-Mei Li

**Affiliations:** Department of Pediatric Intensive Care Unit, The First Hospital of Jilin University, Changchun, Jilin, China.

**Keywords:** 3β-hydroxy-Δ5-C27-steroid dehydrogenase deficiency, HSD3B7, systematic review, prognosis

## Abstract

**Objectives:**

3β-hydroxy-Δ5-C27-steroid dehydrogenase deficiency is a rare autosomal recessive condition. So far fewer than 100 cases have been reported and the factors affecting the prognosis are not yet established. The objective of this study is to explore a possible prediction of the outcome of this rare condition.

**Methods:**

This review was undertaken and reported in accordance with the preferred reporting items for systematic review and meta-analyses guidelines. Demographics, clinical features, gene data, treatment strategies and prognoses at the last follow-up were extracted and summarized. Patients were divided into 2 groups (alive with native liver and liver transplantation/died). Risk factors for the different clinical features were identified.

**Results:**

87 patients that were taken from 7 case reports and 9 case series were included. 38 (38/63, 63.0%) of them presented initial symptoms when they were younger than 1 month and 55 (55/63, 87.3%) less than 1 year. There is a larger proportion of patients younger than 1 month or 1 year at the age of symptom onset in the liver transplantation /died group than patients in alive with the native liver group. The majority of patients (53/62, 85.5%) were diagnosed before the age of 5 year. In all cases, 65 (predicted) pathogenic variants have been identified. Over 70% of patients carried an HSD3B7 variant on exon 1, 4, 5 or 6. 71 (81.6%) were alive at the last follow-up, 16 (18.4%) underwent liver transplantation or died. No significance was found between the group alive with native liver and group liver transplantation /died.

**Conclusion:**

Age of onset of the symptoms may be a potential factor that determines the outcome of patients with 3β-HSD deficiency, patients presented with symptoms and signs at an age younger than 1 month or even 1 year may have a worse prognosis. Since there is no difference between clinical outcome and zygosity of gene mutation, we recommend a further study about any possible relationship between mutation site and clinical characteristics or prognosis.

## 1 Introduction

Inborn errors of primary bile acid (BA) synthesis disorder is a rare autosomal recessive disease, accounting for 1% to 2% of unexplained cholestasis in newborns and children.^[1]^ The deficiency of 3β-hydroxy-Δ^5^-C_27_-steroid dehydrogenase (3β-HSD) due to mutations in gene HSD3B7, is the most common type of primary BA synthesis disorder.^[2]^ The prevalence of 3β-HSD deficiency could be estimated as 1 case per 10 million.^[3]^ Affected individuals show cholestasis, hepatomegaly, splenomegaly and fat (-soluble vitamins) malabsorption, with or without pruritus. Elevated aminotransferase, hyperbilirubinemia, normal or low serum γ-glutamyltransferase levels as well as BA concentration are the characteristic lab findings in most patients with 3β-HSD deficiency.^[^4^,^^5^^]^

Bile acids (Bas) are synthesized from cholesterol through a complex series of reactions. The reactions involve at least 2 pathways (classic and acidic pathways) and 17 different enzymes preferentially expressed in the liver. The classic pathway is predominant in adults, while the acidic pathway plays an important role in neonates.^[6]^ The HSD3B7 gene located on 16p11.2 of the chromosome contains 6 coding exons and encodes 369 amino acids.^[7]^ 3β-HSD encoded by HSD3B7 catalyzes key reactions in the initial stages of the synthesis of primary BAs from cholesterol. Cholic acid (CA) and chenodeoxycholic acid (CDCA), the immediate products of these steps, are the 2 primary BAs that are critical for bile formation.^[6]^ Normal BA provides the primary driving force for the elimination of cholesterol, as well as the endogenous and exogenous toxic substances such as bilirubin and drug metabolites from the body. In the gastrointestinal tract, BA facilitates the absorption of fat and fat-soluble vitamins (A, D, E, K). Abnormal BA is not well transported by canalicular transporters, causing direct liver injury and/or recruitment of inflammatory factors. Abnormal BA will also damage the secretory function and two-way flow of the liver, and ultimately result in cholestasis.^[4]^ The deficiency of 3β-HSD due to the mutation of HSD3B7 will result in the marked reduction or complete lack of CA and CDCA and the accumulation of hepatotoxic atypical BAs, causing reduced bile flow and subsequent malabsorption of fat-soluble vitamins as well as the progression of liver injury and cirrhosis.

To date, several cases of 3β-HSD deficiency diagnosed in childhood have been reported, but the relationship between prognosis and variable clinical characteristics is relatively under-explored. Therefore, we performed a systematic review of cases of 3β-HSD deficiency, aiming to find the potential factors that affect the prognosis.

## 2 Methods

This systematic review was conducted in accordance with the Preferred Reporting Items for Systematic Reviews and Meta-Analyses guidelines (“http://www.prisma-statement.org/”).

### 2.1 Search strategy and selection criteria

Studies were identified using the following databases: MEDLINE, PubMed and Web of Science. The terms used in the search include “3-Hydroxysteroid Dehydrogenases/deficiency,” “primary bile acid synthesis disorder,” “congenital bile acid synthesis disorder,” “HSD3B7,” “3β-hydroxy-Δ^5^-C_27_-steroid dehydrogenase deficiency,” “3β-HSD deficiency,” “3 beta-HSD deficiency,” “3 beta-hydroxy-delta 5-steroid dehydrogenase/isomerase deficiency,” “3 beta-hydroxy-delta 5-C27-steroid dehydrogenase/isomerase deficiency.” The search was limited to peer-reviewed articles published between January 1, 1980 and October 31, 2021. Only articles which recruited patients with 3β-HSD deficiency and were published in English were included. The studies were excluded if cases were diagnosed when they were over 18 years. The reference lists of articles that met the inclusion criteria were scanned for additional publications (snow ball method) that could be candidates for inclusion in the systematic review.

### 2.2 Selection criteria

1. case reports or case-series of 3β-HSD deficiency;
2. diagnosed age ≤18 years;
3. published in English language;
4. full text is available;
5. detailed information was documented.

### 2.3 Data extraction

Microsoft Excel software was used for data extraction. The following information was extracted from each included study: demographics, clinical features, gene data, treatment strategies and prognoses at the last follow-up. Some cases were reported repeatedly, we extracted all the useful information from different articles, the information of a case may come from more than 1 article. Two researchers independently extracted the data. Disagreements between reviewers were resolved through discussion until consensus was reached. Family history is defined as siblings with a liver disease highly suspicious of a primary BA synthesis disorder.

### 2.4 Data description and statistical analysis

Because of the differences in the description of clinical characteristics reported by the included studies, we could only document the original data in accordance with the articles without integration or modification. Qualitative data were presented as numbers and percentages. Quantitative data were presented as the mean with median and interquartile range (IQR). Cases were classified into 2 groups based on the prognosis at the last follow-up, the first group included patients who were alive with native liver, the second group included patients who were performed liver transplantation (LT) or died. Given the heterogeneity and dismissal of original data, a statistical analysis was not conducted for all items. Categorical variables were assessed by the Chi-Squared test or Fischer exact test. All statistical analyses were conducted in SPSS (version 24) and all *P* values were two-tailed, with *P* = .05 considered to be significant.

## 3 Results

**Figure 1:**
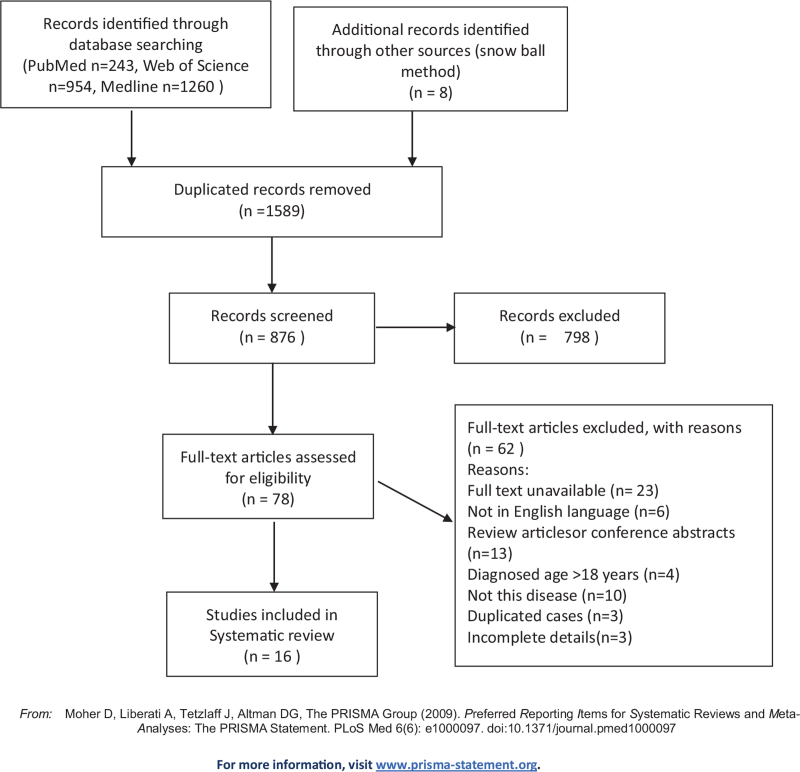
Identification and Selection of Studies for the Systematic Review.

A flowchart detailing the selection of publications is shown in Figure 1. The search of electronic databases yielded 2465 records, among which 1589 were duplicates. Of the remaining 876 records, 78 publications were retained after the title and abstract screening. Ultimately, 16 articles met the inclusion criteria after full-text eligibility assessment.

### 3.1 Overview of studies

We report findings from 87 patients that were taken from 7 case reports and 9 case series.^[^7^,^^8^^–^15^,^^17^^–^23^]^ Table 1 presents detailed characteristics of each case and Table 2 shows the summary of the data. A statistical analysis based on the prognosis of the 87 cases is presented in Table 3.

**Table 1:** Characteristics of all published cases of 3β-HSD deficiency diagnosed at children.

Case no. (Family)	Nationality	Sex	Consanguinity/Family history	Age at diagnosis/Age at onset	Time between age onset and diagnosis	Symptoms
1(A)	Saudi Arabian	M	Y/Y	3 m/3 d	2.9 m	Cholestasis, hepatomegaly, rickets, failure to thrive, pruritus
2(A)	Saudi Arabian	F	Y/Y	died before diagnosis/7 d	NA	Cholestasis, fat-soluble vitamin deficiency, steatorrhea
3(A)	Saudi Arabian	M	Y/Y	died before diagnosis/NA	NA	Cholestasis, fat-soluble vitamin deficiency, steatorrhea, rickets
4(B)	Portuguese	M	Y/Y	4 y/0.3 y	44.4 m	Cholestasis, hepatomegaly, steatorrhea, rickets
5(C)	Chilean	F	Y/Y	4 y/0.1 y	46.8 m	Cholestasis, hepatomegaly, splenomegaly, steatorrhea, renal cyst, areflexia
6(D)	Portuguese	F	Y/Y	7.8 y/3.9 y	46.8 m	Cholestasis, hepatomegaly, fat-soluble vitamin deficiency, areflexia
7(E)	Japanese	M	Y/Y	18 m/1 m	17 m	Cholestasis
8(F)	Asian	F	Y/Y	3 y/9 m	27 m	Cholestasis, hepatomegaly, fat-soluble vitamin deficiency, osteopenia
9(G)	Jordanian	M	Y/Y	3 y/7 m	27.6 m	Fat-soluble vitamin deficiency, failure to thrive, pruritus, steatorrhea
10(H)	French	M	Y/N	2 y/1.75 y	3 m	Cholestasis, hepatomegaly, steatorrhea, renal cyst, areflexia
11(I)	French	F	Y/Y	2.3 y/2 y	3.6 m	Cholestasis, hepatomegaly, splenomegaly, steatorrhea, areflexia
12(I)	French	F	Y/Y	11.5 y/2.4 y	109.2 m	Cholestasis, hepatomegaly, steatorrhea, areflexia, bleeding (vitamin K deficiency)
13(J)	French	F	N/N	0.3 y/0.2 y	1.2 m	Cholestasis, hepatomegaly, steatorrhea
14(K)	British	M	N/N	4.2 y/NA	NA	Rickets, failure to thrive
15(L)	Canadian	F	N/N	13.5 y/NA	NA	Cholestasis, hepatomegaly, splenomegaly, liver cirrhosis
16(M)	African-American	M	N/N	0.6 y/NA	NA	Cholestasis, hepatomegaly, splenomegaly, liver cirrhosis
17(N)	Italian	F	Y/Y	4.3 y/0.5y	45.6 m	Cholestasis, hepatomegaly, splenomegaly, renal cyst, fat-soluble vitamin deficiency
18(N)	Italian	M	Y/Y	NA/0.4 y	NA	Cholestasis, hepatomegaly, steatorrhea, hypocalcemia
19(O)	French	F	Y/Y	4.8 y/0.9 y	46.8 m	Hepatomegaly, steatorrhea, areflexia
20(O)	French	F	Y/Y	13 y/0.75 y	147 m	Hepatomegaly, splenomegaly, renal cyst, steatorrhea, rickets, bleeding (vitamin K deficiency)
21(P)	France-Senegal	M	N/Y	NA/0.5 y	NA	Cholestasis, hepatomegaly, renal cyst, fat-soluble vitamin deficiency, steatorrhea, hypocalcemia
22(P)	France-Senegal	F	N/Y	NA/1 y	NA	Cholestasis, hepatomegaly, fat-soluble vitamin deficiency, steatorrhea
23(Q)	Japanese	M	NA/NA	6 m/1 m	5 m	Cholestasis
24(R)	Italian	M	NA/Y	2.5y/2.5m	27.5 m	Cholestasis, hepatomegaly
25(R)	Italian	M	NA/Y	Screened at birth/asymptomatic	NA	–
26(S)	Moroccan	F	NA/Y	screened at birth/asymptomatic	NA	–
27(S)	Moroccan	M	NA/Y	3 y/ < 1 y	24 m	Cholestasis, hepatomegaly, splenomegaly, failure to thrive
28(S)	Moroccan	F	NA/Y	5.5 y/ < 1 y	54 m	Cholestasis, hepatomegaly, bleeding (vitamin K deficiency)
29–46(NA)	6White/6Arab/4Asia/1Lebanese/1Turkish	12M/6F	6/NA	0.2–11 y/NA(2asympatic)	NA	Cholestasis(n = 11), rickets (n = 8), hepatomegaly (n = 7), pruritus (n = 3), and steatorrhea, and failure to thrive(n = 3), fat-soluble vitamin deficiency (n = 10)
47(T)	Chinese	M	NA/NA	1 m/2 d	0.9 m	Cholestasis
48(U)	Italian	M	N/NA	32 m/1–2 y	17 m	Hepatomegaly, renal cyst, failure to thrive
49(NA)	Chinese	M	NA/NA	5.7 m/1.5 m	4.2 m	Cholestasis, hepatomegaly
50(NA)	Chinese	M	NA/Y	16.5 m/10d	16.2m	Cholestasis, hepatomegaly, renal cyst
51(NA)	Chinese	M	NA/NA	4.5 m/5d	4.3 m	Cholestasis, hepatomegaly
52(NA)	Chinese	F	NA/NA	4.5 m/7 d	4.3 m	Cholestasis, hepatomegaly
53(NA)	Chinese	M	NA/NA	3.7 m/5 d	3.5 m	Cholestasis, hepatomegaly
54(NA)	Chinese	M	NA/Y	17.2 y/16.8 y	4.8 m	Cholestasis, hepatomegaly, splenomegaly, renal cyst, iver failure
55(NA)	Chinese	M	NA/NA	2.2 y/1 m	25.4 m	Cholestasis, hepatomegaly
56(NA)	Chinese	F	NA/NA	4.3 y/3.5y	9.6 m	Coagulopathy of vitamin K deficiency, hepatomegaly, splenomegaly
57(NA)	Chinese	M	NA/NA	6.6 m/1 m	5.6 m	Cholestasis, hepatomegaly
58(NA)	Chinese	M	NA/NA	3.4 y/2–3 d	40.7 m	Cholestasis, hepatomegaly, splenomegaly
59(NA)	Chinese	M	NA/NA	5.2 y/2 d	62.4 m	Cholestasis, hepatomegaly
60(NA)	Chinese	F	NA/NA	2.6 m/1.5 m	1.1 m	Cholestasis
61(NA)	Chinese	F	NA/NA	2 m/10 d	1.7 m	Cholestasis, hepatomegaly
62(NA)	Chinese	M	NA/NA	6.3 m/2 m	4.3 m	Cholestasis
63(NA)	Chinese	F	NA/NA	6.6 y/3 d	79.1 m	Cholestasis, splenomegaly
64(NA)	Chinese	F	NA/NA	5.8y/3d	69.6 m	Cholestasis
65(NA)	Chinese	F	NA/NA	4.8 y/2 m	55.6 m	Cholestasis, hepatomegaly
66(NA)	Chinese	F	NA/NA	4.6 m/1 m	3.6 m	Cholestasis
67(NA)	Chinese	M	NA/NA	1.7 m/3 d	1.6 m	Cholestasis
68(NA)	Chinese	M	NA/NA	5.5 m/2 d	5.4 m	Cholestasis, hepatomegaly, splenomegaly
69(NA)	Chinese	M	NA/NA	11.5 m/10 d	11.2 m	Cholestasis, hepatomegaly, splenomegaly, liver failure
70(NA)	Chinese	M	NA/NA	4.9 m/3–4 d	4.8 m	Cholestasis, hepatomegaly, splenomegaly, liver cirrhosis
71(NA)	Chinese	M	NA/NA	8.7 m/1 m	7.7 m	Cholestasis
72(NA)	Chinese	M	NA/Y	2.4 m/11 d	2 m	Cholestasis, hepatomegaly, splenomegaly
73(NA)	Chinese	M	NA/NA	3 m/3 d	2.9 m	Cholestasis, hepatomegaly, splenomegaly
74(NA)	Chinese	M	NA/Y	2.2 m/1 m	1.2 m	Cholestasis
75(NA)	Chinese	M	NA/NA	2.2 m/7 d	2 m	Cholestasis
76(NA)	Chinese	M	NA/NA	8 m/18 d	7.4 m	Cholestasis
77(NA)	Chinese	M	NA/NA	4.6 m/3 d	4.5 m	Cholestasis
78(NA)	Chinese	M	NA/NA	7.8 m/7 d	7.6 m	Cholestasis
79(NA)	Chinese	F	NA/NA	5.2 y/4 y	14.4 m	Splenomegaly, liver cirrhosis
80(NA)	Chinese	F	NA/NA	3.3 m/3 d	3.2 m	Cholestasis
81(NA)	Chinese	F	NA/NA	5 m/3 d	4.9 m	Cholestasis
82(NA)	Chinese	M	NA/NA	1.8 m/3 d	1.7 m	Cholestasis
83(NA)	Chinese	F	NA/NA	4.7 y/4.5 y	2.4 m	Splenomegaly, liver cirrhosis
84(NA)	Chinese	M	NA/NA	4.4 m/1 m	3.4 m	Cholestasis
85(NA)	Chinese	M	NA/NA	1.8 m/1 m	0.8 m	Cholestasis, liver failure
86(NA)	Chinese	F	NA/NA	4 m/3 d	3.9 m	Cholestasis
87(NA)	Chinese	F	NA/NA	4.7 m/2 d	4.6 m	Cholestasis

Case no. (Family)	MS diagnosis/Gene analysis	Mutation	Zygosity	Oral bile acid replacement treatment before diagnosis	Oral bile acid replacement treatment after diagnosis	Outcome at last follow-up	Reference
1(A)	Y/Y	Exon6, c.del1057CT	Hom	–	CDCA (18 mg/kg/d)	Alive	9–12
2(A)	N/Y	Exon6, c.del1057CT	Hom	–	–	Died following gastrointestinal hemorrhage (19m)	9
3(A)	N/Y	Exon6, c.del1057CT	Hom	–	–	Died from hemorrage following the biopsy(3y9m)	9
4(B)	Y/Y	Exon6, c.1024delT	Hom	UDCA (15 mg/kg/d)	CA	Alive	7, 13
5(C)	Y/Y	Exon2, c.292insC	Hom	UDCA (15 mg/kg/d)	CA	Alive	7, 13
6(D)	Y/Y	Exon1, c.16C > T	Hom	UDCA (15 mg/kg/d)	CA	Alive	7, 13
7(E)	Y/Y	Exon2, c.314delA	Hom	UDCA (12.5 mg/kg/d)	CDCA (8.3 mg/kg/d)	Alive	14
8(F)	Y/N	–	–	–	CDCA (12 mg/kg/d)	Alive	15
9(G)	Y/N	–	–	–	CDCA (9 mg/kg/d)	Alive	15
10(H)	Y/Y	Exon4, c.439G > A	Hom	–	CA	Alive	7
11(I)	Y/Y	Exon4, c.439G > A	Hom	–	CA	Alive	7
12(I)	Y/Y	Exon4, c.439G > A	Hom	–	CA	Alive	7
13(J)	Y/Y	①Exon1, c.114insCC ②Exon1,c.73delGTG	Het	–	CA	Alive	7
14(K)	Y/Y	Exon1, c.60delAG	Hom	–	CA	Alive	7
15(L)	Y/Y	Exon1, c.60delAG	Hom	–	CA	LT	7
16(M)	Y/Y	①Exon1, c.G19S, ②Exon1, c.140delTC	Het	–	UDCA + CA	LT	7
17(N)	Y/Y	Exon2, c.292insC	Hom	UDCA (15 mg/kg/d)	CA	Alive	16
18(N)	Y/Y	Exon2, c.292insC	Hom	–	CA	Alive	16
19(O)	Y/Y	Intron5, c.695-1G > A	Hom	UDCA (15 mg/kg/d)	CA	Alive	16
20(O)	Y/Y	Intron2, c.322 + 1G > T	Hom	UDCA (15 mg/kg/d)	CA	Alive	16
21(P)	Y/Y	①Exon1, c.45-46delAG ②Intron3,c.432-2delA	Het	–	CA	Alive	17
22(P)	Y/Y	①Exon1, c.45-46delAG ②Intron3, c.432-2delA	Het	–	CA	Alive	17
23(Q)	Y/Y	①Exon3, c.412G > T ②Exon4,c.464C > T ③Exon6,c.973-974insCCTGC	Het	UDCA (19 mg/kg/d)	CDCA (15.6 mg/kg/d)	Alive	18
24(R)	Y/N	–	–	UDCA	UDCA (5 mg/kg/d) + CDCA(5mg/kg/d)	Alive	19
25(R)	Y/N	–	–	–	UDCA (7.5 mg/kg/d) + CDCA(7.5mg/kg/d)	Alive	19
26(S)	Y/N	–	–	–	UDCA (10 mg/kg/d) + CDCA(10mg/kg/d)	Alive	19
27(S)	Y/N	–	–	–	UDCA (5 mg/kg/d) + CDCA(5 mg/kg/d)	Alive	19
28(S)	Y/N	–	–	–	UDCA (5 mg/kg/d) + CDCA(5 mg/kg/d)	Alive	19
29–46(NA)	Y/N	–	–	NA	7[CA (7 mg/kg/d) + CDCA(7 mg/kg/d)] 7CDCA(7-18 mg/kg/d) 1CA(4-18 mg/kg/d) 3 died before treatment	15Alive 3Died	20
47(T)	N/Y	①Exon5, c.503G > A ②Exon6, c.683G > A	Het	UDCA	CDCA	Alive	21
48(U)	Y/Y	Exon3, c.432-2A > G	Hom	UDCA (10 mg/kg/d)	CA (12 mg/kg/d)	Alive	22
49(NA)	Y/Y	Exon6, c.1031A > G	Hom	NA	UDCA + CDCA	Alive	23
50(NA)	Y/Y	①Exon1, c.45-46delAG ②Exon6, c.988-990delACC	Het	NA	UDCA + CDCA	Alive	23
51(NA)	N/Y	Exon6, c.968C > T	Hom	NA	NA	Alive	23
52(NA)	Y/Y	①Exon5, c.683G > A ②Exon6, c.1040delT	Het	NA	NA	Died	23
53(NA)	Y/Y	①Exon1, c.45-46delAG ②Exon2, c.262G > C	Het	NA	CDCA	Alive	23
54(NA)	N/Y	Exon4, c.484-485delinsCC	Hom	NA	NA	Died	23
55(NA)	Y/Y	Exon5, c.544delC	Hom	NA	NA	Alive	23
56(NA)	Y/Y	Exon4, c.474delC	Hom	NA	CDCA	Alive	23
57(NA)	Y/Y	①Exon5, c.543dupG ②Exon6, c.790C > A	Het	NA	CDCA	Alive	23
58(NA)	Y/Y	①Exon6, c.781G > A ②Exon6, c.1079G > A	Het	NA	CDCA	Alive	23
59(NA)	Y/Y	①Exon3, c.401G > A ②Intron4, c.532-3 C > G	Het	NA	CDCA	Alive	23
60(NA)	Y/Y	①Exon5, c.682 C > T ②Exon6, c.1061G > C	Het	NA	CDCA	Alive	23
61(NA)	Y/Y	①Exon4, c.503G > A ②Exon5, c.683G > A	Het	NA	CDCA	Alive	23
62(NA)	Y/Y	①Exon1, c.147G > A ②Exon4, c.503G > A	Het	NA	CDCA	Alive	23
63(NA)	Y/Y	①Exon4, c.503G > A ②Exon5,c.569G > A	Het	NA	CDCA	Alive	23
64(NA)	Y/Y	Exon5, c.682C > T	Hom	NA	CDCA	Alive	23
65(NA)	Y/Y	Exon6, c.988-990delACC	Hom	NA	CDCA	Alive	23
66(NA)	Y/Y	①Exon5, c.543dupG ②Exon5, c.683G > A	Het	NA	CDCA	Alive	23
67(NA)	Y/Y	①Exon1, c.45-46delAG ②Exon6, c.770 A > G	Het	NA	CDCA	Alive	23
68(NA)	Y/Y	①Exon5, c.683G > A ②Exon5, c.683G > T	Het	NA	CDCA	Alive	23
69(NA)	Y/Y	①Exon5, c.561T > G ②Exon5, c.586G > A	Het	NA	CDCA	LT	23
70(NA)	Y/Y	①Exon3, c.346T > C ②Exon6, c.964-965dup	Het	NA	CDCA	Alive	23
71(NA)	Y/Y	Exon4, c.503G > A	Hom	NA	UDCA	Alive	23
72(NA)	Y/Y	①Exon5, c.676 C > T ②Exon5, c.-205_323-108del	Het	NA	CDCA	Alive	23
73(NA)	Y/Y	①Exon4, c.503G > A ②Exon6, c.743G > C	Het	NA	CDCA	LT	23
74(NA)	Y/Y	Intron3, c.431 + 2dupT	Hom	NA	CDCA	Alive	23
75(NA)	Y/Y	Exon4, c.485-487delGCA	Hom	NA	CDCA	Alive	23
76(NA)	Y/Y	①Exon5, c.683G > A ②Intron5, c.694 + 2T > C	Het	NA	CDCA	LT, died	23
77(NA)	Y/Y	①Exon2, c.173-174del ②Exon3, c.371T > C	Het	NA	CDCA	Alive	23
78(NA)	Y/Y	①Exon5,c.557 C > T ②Exon6,c.968 C > G	Het	NA	CDCA	LT	23
79(NA)	Y/Y	①Exon1, c.45_46delAG ②Exon5, c.543dupG	Het	NA	CDCA	Alive	23
80(NA)	Y/Y	Exon4, c.499G > A	Hom	NA	CDCA	Alive	23
81(NA)	Y/Y	①Exon6, c.698 A > G ②Exon6, c.1033G > T	Het	NA	CDCA	Alive	23
82(NA)	Y/Y	①Exon6, c.920-931delGGCTGC TGCGGC ②Exon5, c.543dupG	Het	NA	CDCA	LT	23
83(NA)	Y/Y	①Exon1, c.45-46delAG ②Exon2, c.319 C > T	Het	NA	CDCA	Alive	23
84(NA)	N/Y	①Exon1, c.45-46delAG ②Exon6, c.905delA	Het	NA	CDCA	Died	23
85(NA)	N/Y	①Exon3, c.402-403insG ②Exon4, c.503G > A	Het	NA	CDCA	Alive	23
86(NA)	N/Y	Exon5, c.543dupG	Hom	NA	CDCA	Alive	23
87(NA)	N/Y	Exon4, c.503G > A	Hom	NA	CDCA	Died	23

**Table 2:** Summary of the characteristics of all cases of 3β-HSD deficiency diagnosed at childhood.

Variables (available data)		N	%
Sex (87)			
	M	53	60.9
	F	34	39.1
Consanguinity (41)			
	Y	22	53.7
	N	19	46.3
Family history (31)			
	Y	26	83.9
	N	5	16.1
Age at onset (63)			
Median:1 m	≤1 m	38	60.3
Range:2 d-16.8 y	>1 m	25	39.7
IQR: 3 d–6 m			
	≤1 y	55	87.3
	>1 y	8	12.7
Age at diagnosis (62)			
Median:8.35m	≤1 y	38	61.3
Range:1 m–17.2 y	>1 y	24	38.7
IQR: 4.4–50.4 m			
	≤5 y	53	85.5
	>5 y	9	14.5
Time between age at onset and diagnosis (59)			
Median:5 m	≤3 m	14	23.7
Range:0.8–147 m	3–6 m	18	30.5
IQR: 3.3–27.6 m	6 m–2 y	10	16.9
	2–5y	12	20.3
	>5 y	5	8.5
Zygosity (62)			
	Hom	30	48.4
	Het	32	51.6
Oral bile acid replacement treatment before diagnosis (11)			
	UDCA	11	100
Oral bile acid replacement treatment after diagnosis (78)			
	CDCA	45	57.7
	CA	17	21.8
	CDCA + UDCA	7	9.0
	CA + CDCA	7	9.0
	UDCA	1	1.3
	CA + UDCA	1	1.3
Outcome (87)			
	Alive with native liver	71	81.6
	LT/Died	16	18.4

^∗^NA: not available.

**Table 3:** Differences between patients alive with native liver and LT/Died.

Variables		Alive with native liver (N = 56)	LT/Died (N = 13)	*P*
Sex	M	32 (57.1%)	9 (69.2%)	.42
	F	24 (42.9%)	4 (30.8%)	
Consanguinity	Y	14 (73.7%)	2 (50.0%)	.56
	N	5 (26.3%)	2 (50.0%)	
	NA	37	9	
Family history	Y	23 (88.5%)	3 (60.0%)	.17
	N	3 (11.5%)	2 (40.0%)	
	NA	30	8	
Age at onset	Range	2 d–4.5 y	2d-16.8y	
	≤1 m	29 (54.7%)	9 (90.0%)	.08
	>1 m	24 (45.3%)	1 (10.0%)	
	NA	3	3	
	≤1y	46 (86.8%)	9 (90.0%)	1.00
	>1y	7 (13.2%)	1 (10.0%)	
	NA	3	3	
Age at diagnosis	Range	1 m–13 y	1.8 m–17.2 y	
	≤1 y	29 (56.9%)	9 (81.8%)	.23
	> 1y	22 (43.1%)	2 (18.2%)	
	NA	5	2	
	≤5 y	44 (86.3%)	9 (81.8%)	1.00
	>5 y	7 (13.7%)	2 (18.2%)	
	NA	5	2	
Time between age at onset and diagnosis	≤6 m	26 (52.0%)	6 (66.6%)	.65
	>6 m	24 (48.0%)	3 (33.3%)	
	NA	6	4	
Zygosity	Hom	25 (51.0%)	5 (38.5%)	.42
	Het	24 (49.0%)	8 (61.5%)	
	NA	7	0	
Oral bile acid replacement treatment before diagnosis	UDCA	11 (100%)	0 (0)	NA
	NA	45	13	
Oral bile acid replacement treatment after diagnosis	CDCA	31 (57.4%)	7 (77.8%)	.67
	CA	15 (27.8%)	1 (11.1%)	
	CDCA + UDCA	7 (12.9%)	1 (11.1%)	
	UDCA	1 (1.9%)	0 (0)	
	NA	2	4	
Cholestasis	Y	46 (92.0)%	13 (100%)	.23
	N	10 (8%)	0 (0)	
Hepatomegaly	Y	29 (51.8%)	6 (46.2%)	.71
	N	27 (48.2%)	7 (53.8%)	
Splenomegaly	Y	13 (23.2%)	5 (38.5%)	.26
	N	43 (76.8%)	8 (61.5%)	
Steatorrhea	Y	12 (21.4%)	2 (15.4%)	.92
	N	44 (78.6%)	11 (84.6%)	
Fat-soluble vitamin Deficiency	Y	6 (10.7%)	2 (15.4%)	1.00
	N	50 (89.3%)	11 (84.6%)	
Rickets	Y	4 (7.1%)	1 (7.7%)	1.00
	N	52 (92.9%)	12 (92.3%)	
Failure to thrive	Y	5 (9.0%)	0 (0)	.58
	N	51 (91.1%)	13 (100%)	
Renal cyst	Y	7 (12.5%)	0 (0)	.93
	N	49 (72.5%)	13 (100%)	
Areflexia	Y	6 (10.7%)	0 (0)	.49
	N	50 (89.3%)	13 (100%)	
Pruritus	Y	2 (3.6%)	0 (0)	1.00
	N	54 (96.4%)	13 (100%)	
Bleeding due to vitamin K deficiency	Y	4 (7.1%)	0 (0)	1.00
	N	52 (92.9%)	13 (100%)	

^∗^NA: not available.

### 3.2 Patient characteristics

There were 53 males (60.9%), with a male-to-female ratio of 1.56/1 (Table 2). Age of onset was reported for 63 cases, the median age was 1 m (IQR: 3 days–6 months, range 2 days–16.8 years). 38 (38/63, 63.0%) of them presented initial symptoms when they were younger than 1 month and 55 (55/63,87.3%) before the age of 1 years. Sixty-two cases were reported age at diagnosis, the median age as 8.35 month (IQR: 4.4–50.4 months, range 1m-17.2y). The majority of patients (53/62, 85.5%) were diagnosed before 5 years. The median time between age at onset of symptoms and diagnosis of 59 cases was 5 m (IQR: 3.3–27.6 months, range 0.8–147 months). Cases of 3β-HSD deficiency were reported worldwide and were more commonly reported in China, France and Arabia. Specifically, the nationalities of cases were African-American, British, Canadian, Chilean, Jordanian, Lebanese, Turkish (each n = 1), France-Senegal, Portuguese, Japanese (each n = 2), Moroccan, Saudi Arabian (each n = 3), Italian, Asian (each n = 5), French and Arab (each n = 6), Chinese (n = 40). In a case series, the specific nationality of 6 cases was not described, the race of them was white (n = 6). Cholestasis was the most observed manifestation (70/87, 80.5%), followed by hepatomegaly (42/87, 48.3%), splenomegaly (18/87, 20.7%), fat-soluble vitamin deficiency (18/87, 20.7%) and steatorrhea (17/87, 19.5%). Unusual manifestations included rickets (13/87, 14.9%), failure to thrive (8/87, 9.2%), renal cyst (8/87, 9.2%), areflexia (6/87, 6.9%), pruritus (5/87, 5.7%) and bleeding due to vitamin K deficiency (4/87, 4.6%). 4 patients who were identified through family screening were still asymptomatic when diagnosed. Consanguinity and family history were not rare. There were 22 patients from consanguineous families and 26 patients had family histories.

### 3.3 Genetic spectrum

Of 62 (62/87, 66.7%) cases, the detail of the gene mutation was described, of these, 30 (30/62, 48.4%) were homozygous mutations, 32 (32/62, 51.6%) were compound heterozygous mutation. There were 65 (predicted) pathogenic variants identified. The variants identified were spread throughout the HSD3B7 gene, specific mutations were listed in Table 1. The variants were in exon 1 (8, 12.3%), exon 2 (5, 7.6%), intro 2 (1, 1.5%), exon 3 (6, 9.2%), intro 3 (2, 3.1%), exon 4 (8, 12.3%), intro 4 (1, 1.5%), exon 5 (12, 18.5%), intro 5 (2, 3.1%), exon 6 (20, 30.8%) separately. Over 70% of patients carried an HSD3B7 variant on exon 1, 4, 5, or 6.

### 3.4 Treatment and outcome

Eleven patients were treated with ursodeoxycholic acid (UDCA) before diagnosis (ranging dose 10–19 mg/kg/d). After diagnosis, 78 patients were treated with oral BA replacement treatment, including CDCA 45 (51.7%), CA 17 (19.5%), CDCA and UDCA 7 (8.1%), CA and CDCA 7 (8.1%), UDCA 1 (1.2%), CA and UDCA 1 (1.2%). A review of clinical outcomes found that 71 (81.6%) were alive at last follow-up, 16 (18.4%) underwent LT or died, of whom 6 patients were conducted LT, 9 patients died, 1 patient died after LT. Associations between patients’ variable clinical features and prognosis are summarized in Table 3. No significance was found between the group alive with native liver and group LT/died. However, compared to the cases alive with native liver, a larger proportion of patients in the LT/died group was fewer than 1 month or 1 year at the age of onset of the symptoms.

## 4 Discussion

This study is the first systematic review of patients with 3β-HSD deficiency diagnosed in childhood reported to date. We divided patients into 2 groups based on prognosis, no significance was found in sex, consanguinity, family history, age at onset, age at diagnosis, the time between age at onset of the symptoms and diagnosis, zygosity, manifestations, and treatment.

According to our results, although there was no statistical difference in the age of onset between the 2 groups, a larger proportion of patients who were less than 1 year old or 1 month old at the onset of this genetic condition was found in the LT/died group. This is consistent with the research of Zhao et al.^[22]^ This may suggest that the clinical spectrum of patients with early-onset was worse than that of patients with later onset. Our statistical analysis found no relationship between any clinical symptoms and outcomes. Some children showed uncommon manifestations such as pruritus, renal cyst and areflexia. There are only 5 patients who had pruritus. Jacquemin E^[12]^ suggested that the inadequacy or absence of primary BA could be related to variable expression of the enzyme defect, and the absence of pruritus may be the result of the failure to synthesize primary BAs. This indicates us we can discover the correction between pruritus and the quantity of BA level in serum and urine at the later study. The mechanisms of renal cyst and areflexia are not clear completely. According to a previous study, areflexia is likely due to vitamin E deficiency.^[15]^ Renal cyst is because the unusual BAs were excreted via the kidneys and high concentrations of BA can be toxic to renal tubules and may generate or initiate renal lesions.^[^22^,^^24^^]^ What’ more, the recognition of this rare condition may be influenced by its description in the regional medical journals and therefore more cases come to attention, which could possibly explain why there are many differences in regions.

In group LT/died, despite all patients (apart 2 cases, cases 19 and 54) were diagnosed and given oral BA replacement treatment when they were fewer than 1 year, the prognoses were poor. However, some cases (case 20) and other reported cases could be alive until adolescence and adult without any BA replacement treatment.^[^16^,^^25^^,^^26^^]^ We speculated that the variable phenotypes conceivably were the consequence of heterogeneous molecular basis. Thus, we analyzed the outcome of patients based on homozygous and heterozygous mutations, however, no difference was found. In the largest study of patients with HSD3B7 deficiency, capital Zhao et al^[22]^ categorized the gene variants into 2 classes: null variants (including frameshift, nonsense, classical splicing variants and large fragment deletions) and non-null variants (including missense, non-classical splicing and non-frameshift small indel types, no significant differences were observed in terms of clinical outcome among the patients with different genotypes. The deviation may be caused by the limited number of patients. Our data showed that over 70% of patients carried an HSD3B7 variant on exon 1, 4, 5, or 6. With more cases are reported in the future, the relationship between mutation sites and prognosis could be a new study orientation.

Recognition of this genetic condition relies on the BA spectrum traditionally in serum and urine to establish an absence or marked reduction in synthesis of the normal primary BA (CA and CDCA), as well as the accumulation of excessive atypical BA such as 3β-hydroxy-Δ5-bile acids. The traditional mass spectrometric (MS) techniques to detect BA profiles include fast atom bombardment-mass spectrometry, liquid secondary ionization mass spectrometry and gas chromatography/mass spectrometry. Fast atom bombardment-mass spectrometry analysis of urine was also used to monitor the therapeutic response to primary BA therapy. Dose adjustments of CDCA were based on the findings of reductions in the levels of atypical 3β-hydroxy-Δ5-bile acids from the urinary BA analyses combined with the serum biochemistries. Though MS is faster and more convenient than gene analysis as well as has the ability to detect the therapeutic response to primary BA therapy, it still has limitations and sometimes can be misleading. For patients who have been treated with BA, the BA profiles are probably normal. Heubi JE^[4]^ suggested the BA spectrum should not be analyzed in patients with cholestasis while they are undergoing UDCA treatment because UDCA is metabolized in early life and can be partially converted to CDCA and CA. In addition, the presence of the 7α-hydroxyl group in atypical BA generates a structure that is highly labile and readily degrades under acidic conditions, which could interfere with the test results by the approach of MS. Therefore, MS should be conducted before any treatment with BA or samples should be collected after stopping BA treatment for 4 to 5 days at least. To sum up, it is necessary to combine genetic analyses with MS to diagnose this genetic condition.

Oral BA replacement treatment consists of monotherapy or the combination of CA, CDCA and UDCA. According to our data, patients are treated in a variety of ways after diagnosis. However, there is no correlation between different treatments and prognoses.

For these 3 BAs, CA and CDCA are thought to be more effective and safer than UDCA. CA and CDCA both supply the primary BA for patients with 3β-HSD deficiency and can target the negative feedback regulation for BA synthesis to reduce the production of hepatotoxic abnormal BAs. Between CDCA and CA, CA is likely to be more acceptable since it is neither hepatotoxic nor embryotoxic/teratogenic. CA is the only primary BA having a marketing authorization in the USA and in Europe and is the first-line therapy for patients with 3β-HSD deficiency.^[^24^,^^27^^]^ One study described the long-term effects of CA. With CA therapy, patients have survived to adulthood with a mean age of 24.3 y (range: 15.3–37.2 year) and a normal quality of life at last follow-up, which lasted 21.4 years by average (range: 14.6–24.1 years). All patients had improved liver dysfunction, freedom from treatment complications, normal growth and development, and absence of psychosocial problems. Especially, there were normal pregnancies during CA therapy in 1 patient. These all suggested CA is an effective, well-tolerant therapy option without hepatotoxic and embryotoxic/teratogenic.^[27]^ Prolonged oral CDCA therapy is considered to be safe and lifesaving as it leads to normalization of clinical features, serum liver biochemistry and liver imaging, together with a substantial improvement of mass spectrometry BA profiles and liver histology.^[24]^ Despite the similar treatment mechanism with CA, CDCA is not recommended when patients have live cirrhosis because it can be hepatotoxic at that stage.^[28]^ Additionally, CDCA is contraindicated during pregnancy. Unfortunately, CDCA is used instead of CA in some areas such as Japan and China, since the latter is not available for clinical use.^[24]^ Although UDCA relieves cholestasis, UDCA is not optimal because it does not reduce and may enhance the production of hepatotoxic abnormal BA. UDCA could improve the liver dysfunction of patients in the short term but not decrease the production of atypical BA.^[^18^,^^29^^]^This is because UDCA competitively inhibits the absorption of other BAs and replaces them in the enterohepatic pool, reducing the effectiveness of negative feedback inhibition on BA synthesis provided by oral CDCA.^[30]^ Thus, UDCA in the long-term treatment is seldom useful and should be stopped and replaced with primary BA.^[^15^,^^31^^,^^32^^]^ Above all, CA is the safest and the most effective treatment option for 3β-HSD deficiency.

There are some limitations of this study. Firstly, we did not list the initial time of treatment of oral BA replacement treatment, so we can not obtain the relation between prognosis and timing of BA replacement treatment. Secondly, the clinical data are not complete though we extracted the detailed information of patients from 1 or more publications. Despite these, this first systematic review of 3β-HSD deficiency diagnosed in children is meaningful to learn the variable clinical features and prognosis.

## 5 Conclusion

The systematic review revealed that the age of onset of the symptoms may be a potential factor that determines the outcome of patients with 3β-HSD deficiency, patients presented symptoms and signs at an age younger than 1 month or even 1 year may have a worse prognosis. Since there is no difference between clinical outcome and zygosity of gene mutation, we recommend a further study about any possible relationship between mutation site and clinical characteristics or prognosis.

## Author contributions

**Conceptualization:** Chun-Feng Yang.

**Data curation:** Yuan Zhang, Chun-Feng Yang, Wen-Zhen Wang, Yong-Kang Cheng.

**Formal analysis:** Yuan Zhang, Chun-Feng Yang, Yumei Li.

**Funding acquisition:** Yumei Li.

**Methodology:** Yuan Zhang, Chu-Qiao Sheng, Yumei Li.

**Software:** Yong-Kang Cheng. Supervision: Chun-Feng Yang, Chu-Qiao Sheng.

**Validation:** Yumei Li.

**Writing – original draft:** Yuan Zhang.

**Writing – review & editing:** Yuan Zhang, Yumei Li.
